# Eight-month-old infants’ behavioral responses to peers’ emotions as related to the asymmetric frontal cortex activity

**DOI:** 10.1038/s41598-018-35219-4

**Published:** 2018-11-21

**Authors:** Maria M. Crespo-Llado, Ross Vanderwert, Elisa Roberti, Elena Geangu

**Affiliations:** 1 Lancaster University Department of Psychology Bailrigg, Fylde College, Lancaster, UK; 2 Cardiff University Centre for Human Developmental Science School of Psychology - Cardiff University Tower Building, Park Place, Cardiff, UK; 3 Università degli Studi di Milano - Bicocca Department of Psychology, Milan, Italy; 4 University of York, Department of Psychology, Heslington, York, UK

## Abstract

Infants are sensitive to and converge emotionally with peers’ distress. It is unclear whether these responses extend to positive affect and whether observing peer emotions motivates infants’ behaviors. This study investigates 8-month-olds’ asymmetric frontal EEG during peers’ cry and laughter, and its relation to approach and withdrawal behaviors. Participants observed videos of infant crying or laughing during two separate sessions. Frontal EEG alpha power was recorded during the first, while infants’ behaviors and emotional expressions were recorded during the second session. Facial and vocal expressions of affect suggest that infants converge emotionally with their peers’ distress, and, to a certain extent, with their happiness. At group level, the crying peer elicited right lateralized frontal activity. However, those infants with reduced right and increased left frontal activity in this situation, were more likely to approach their peer. Overall, 8-month-olds did not show asymmetric frontal activity in response to peer laughter. But, those infants who tended to look longer at their happy peer were more likely to respond with left lateralized frontal activity. The link between variations in left frontal activity and simple approach behaviors indicates the presence of a motivational dimension to infants’ responses to distressed peers.

## Introduction

Toddlers’ relationships with their peers have many positive developmental outcomes in later years, including higher levels of emotional mental health and school success^1–4^. Their ability to empathize with their peers, to comfort and to share toys with them increases their chances of becoming friends or preferred play partners. Thus, in order to foster adaptive social development, it is important to understand the development of factors contributing to establishing relations with peers, such as empathy and prosocial behaviors^5–7^. Accumulating evidence suggests that before their first birthday, infants are sensitive and respond to their peers’ emotions, which could represent potential precursors of empathy and prosocial behaviors^8–11^. However, the neurocognitive mechanisms underlying these early responses and the relation between them remains unclear. In order to address this gap, the current study investigates infant asymmetric frontal EEG alpha power in response to peers’ affective states and its relation to simple behavioral manifestations of social approach and withdrawal.

Empathy and prosocial behavior are multifaceted constructs. Empathy is an affective response triggered by and congruent with others’ emotions, regulated to a certain extent and accompanied by some minimal implicit distinction between the self and the other^12,13^. As the definition implies, a multitude of processes are involved in generating empathy, from the perception, evaluation and understanding of others’ emotions, to emotional reactivity and regulation, as well as self-awareness^12–14^. In the same vein, the repertoire of behaviors that can ultimately contribute to the welfare of others are heterogeneous (e.g., comforting, helping, sharing resources) and vary in the extent to which they tap on our cognitive and motor abilities to understand, plan and implement complex sequences of coordinated motor acts^15^. When empathy is associated with the motivation to act, it can lead to the manifestation of prosocial behaviors^16^. The processes underlying the many facets of empathy and prosocial behaviors rely on relatively separable neurophysiological systems. From an ontogenetic point of view, this is particularly relevant, because these neuropsychological systems have different developmental trajectories^8,10^, which could influence the characteristics of different precursors of empathy and prosocial behaviors, and their relation during early development^8,10,17,18^.

Throughout the first year of life, infants respond emotionally to various cues of their peers’ affective states. At birth, neonates cry when they hear another human neonate crying^19–23^. These emotionally convergent responses to their peers’ cry persist throughout infancy and toddlerhood, yet with lesser intensity^24,25^. At the psychophysiological level, changes in arousal can also be observed shortly after the onset of peers’ crying, while overt emotional expression cues are usually observed after prolonged stimulation^24,26–30^. The more infants are able to use self-soothing behaviors and attentional strategies to regulate their emotions, the more likely they are to down-regulate these negative affect sharing responses^26^. Around the age of 9-months, infants who have greater abilities to discriminate perceptually between their own body and that of another infant, tend to show less intense emotional responses to their peers’ cry^26^. Thus, besides being congruent with the observed emotion, these responses are also related to infants’ abilities to regulate their own emotions and to differentiate between self and others, raising the possibility that they could be precursors of empathy development. Although less investigated but equally important, infants also seem tuned to their peers’ positive affect. Watching video recordings of peers laughing was shown to elicit increases in the level of infants’ arousal as indexed by changes in the pupil diameter^28,29^. Both younger (6-month-old) and older (12- and 15-month-old) infants show such increases in arousal; however, in younger infants these responses appear after longer latencies and are less persistent in time compared to older infants. Moreover, as the infants reach their first birthday, the arousal elicited by peers’ emotions becomes more negatively biased, with negative emotions eliciting a higher arousal compared to the positive ones^28,29^. Neuroimaging investigations using fMRI and ERP methods indicate that infants’ affective responses to others’ emotions rely on neurocognitive mechanisms which develop throughout infancy in relation to an increasing cortical specialization for processing human faces^31^ and voices^32,33^, as well as the activity of brain areas known to be involved in the automatic appraisal of the emotional stimuli and the generation of emotional experiences (e.g., the orbitofrontal cortex, the insula^33,34^).

Before the age of one, infants do not only react emotionally to their peers’ affective states, but also engage with them in simple forms of interaction involving behaviors within their motor-repertoire. For example, Vandell and Wilson^35^ showed that the interactions between infants become increasingly reciprocated from 6- to 9-months, as reflected by the presence of turn taking. Looking at the peer with or without associated vocalizations, moving towards the peer, touching the body of the peer or the toy she holds, as well as gesture-like movements were the type of behaviors that efficiently supported the reciprocated social engagement^35,36^. Interestingly, these behaviors have also been recorded during naturalistic interactions in response to peers’ distress^30^. The simple social approach behaviors described to support efficient interactions between infants are the foundation for any complex prosocial behaviors. In order to comfort, one needs to approach, to touch, and potentially vocally communicate with those in distress. In toddlerhood and pre-school years, these simple approach behaviors are important dimensions of children’s reactions to their peers’ distress, alongside empathic responses and other motorically-complex prosocial behaviors^17,18,37^. It is thus possible that the simple social approach behaviors present in infancy are also the precursors of the later developing complex prosocial manifestations. Although some previous studies have explored infants’ emotional responses (e.g., fear, sadness, crying) and attempts to help and comfort their peers, no significant concurrent relations were observed at the age of 8–10-months^38^. One possible interpretation of these findings is that infants’ emotional responses towards their peer’s affect do not translate into overt behavioral actions at this age. It is also possible that the overreliance on recording the more complex prosocial behaviors and the overt manifestations of emotional responses was not sensitive enough to capture possible precursors of prosocial tendencies. Moreover, observational methods are also opaque in terms of the neurocognitive processes underlying different dimensions of infants’ responses to their peers’ emotions, and, as a consequence, less sensitive in detecting the relation between them^39,40^. Research with adults has shown that watching images of people expressing positive and negative affect activates both the neural networks involved in experiencing those emotions and the septal area brain region functionally associated with the motivation to act pro-socially^16,41–43^. The level of activation of the septal area while observing others’ emotions, but not other regions of the emotional network, specifically predicted how frequently participants behaved prosocially in daily life. These results support the idea that there is a motivational dimension to empathic responsivity, which is relevant for the occurrence of pro-social behaviors, and that the generic emotional response may not always be indicative of whether someone will behave prosocially. Developmental social neuroscience investigations sensitive to this motivational dimension are thus useful for understanding the origins of infants’ other-oriented behaviors elicited by peers’ emotions.

The study of the asymmetric frontal EEG alpha power in response to peers’ emotions has the potential to give insights into the presence of a motivational correlate to infants’ social approach behaviors. The approach-withdrawal motivational model of frontal asymmetry relies on the assumption that differences in the EEG alpha power between homologous right and left frontal electrodes reflects the activity of the frontal cortex^44,45^. According to this model, the activity of the left frontal cortex is related to appetitive motivation, the motivation to act towards achieving a goal, and approach-related affect such as happiness, while the activity of the right frontal cortex is associated with withdrawal, behavioral inhibition, and vigilant attention which typically occurs during some negative affective states such as fear and sadness^45–50^. Indeed, infants usually show a left lateralization of the frontal EEG alpha power (i.e., greater activity in the right hemisphere) during social and non-social situations which also elicit facial and vocal expression of negative affect: when an adult stranger approaches while the mother is absent^51,52^, when infants observe adults’ facial expressions of pain^53,54^ and sadness^54,55^, or when confronted with scary toys (e.g., masks, spiders^51^). Importantly, the right lateralization of the frontal EEG alpha power was also found to significantly predict the manifestation of withdrawal behaviors in these situations, including moving away from the source of distress^51^. The right lateralization of the frontal EEG alpha power (i.e., greater activity in the left hemisphere) was recorded in infants in response to adult expressions of happiness or the presence of a familiar caregiver^52–56^, and predicted simple approach behaviors like vocalizations directed towards the other^54,57^. Considering the importance of developing adaptive relations with peers, the current study aims to investigate the extent to which the pattern of infant asymmetric frontal EEG alpha power recorded previously in response to adult emotional experiences extends to their peers’ affective states, and relates to simple behavioral manifestations of social approach and withdrawal. Given the limited evidence about how infants’ responses towards their peers vary as a function of the valence of the observed emotions, both positive (i.e., laughter) and negative (i.e., crying) emotions were included in the study.

Eight-month-old infants were presented with video recordings of other infants laughing and crying during two separate testing sessions. One session was set-up to facilitate the recording of the EEG. In light of previous findings, we anticipated that observing a peer crying will elicit increased left lateralized EEG alpha power relative to the right hemisphere, while observing a peer laughing the opposite pattern, suggesting increased right and left, respectively, frontal cortex activity. Considering the potential of perceiving another’s distress as motivating social approach, we also expected to observe variations in the degree of lateralization to the right of the frontal EEG alpha power in response to the crying infant video. Individual variability in the lateralization of the frontal EEG alpha power was anticipated to relate significantly to the manifestation of the simple approach behaviors and expressions of emotion. In order to facilitate the manifestation of such responses, infants were presented with video recordings of peers laughing and crying in a separate behavior-only session, in the absence of the constraints required for artifact-free EEG recording, such as reduced body movement. We predicted that those 8-month-old infants who responded with increased right lateralization of the frontal EEG alpha power to their peers’ positive and negative emotions would be more likely to approach them behaviorally. The second session also gave us the opportunity to provide a more detailed account of the types of behavioral responses elicited by peers’ positive emotions in 8-month-old infants.

## Methods

### Participants

Forty 8-month-old infants participated in this study. Out of this sample, 32 infants (15 females, *M*_*age*_ = 254.16 days, *SD*_*age*_ = 9.36 days) provided analyzable data for the EEG recording (Session 1) based on the criteria described below. For the behavioral recording (Session 2), 22 infants (13 females, *M*_*age*_ = 254.45 days, *SD*_*age*_ = 9.68 days) provided analyzable data based on the criteria described below. Eighteen infants (12 females, *M*_*age*_ = 252.61 days, *SD*_*age*_ = 8.93 days) contributed analyzable data for both EEG and behavioral recordings. From the sample participating in Session 1, 9 participants did not return to the lab for Session 2 because the parents found it difficult to fit another visit into their schedule. More information about attrition rates for each session is presented in the following sections. All participants were recruited from a small urban area in North West England, did not suffer from any neurological or other medical condition, and were observed to have normal vision and audition for their age.

Prior to both sessions, all parents were informed that at the end of the experiment they would receive £10 in order to cover traveling expenses and that the infant will be rewarded with a book for their participation. Informed consent was obtained from all parents prior to the beginning of the procedure. The procedure was carried out in accordance with the ethical standards of the Declaration of Helsinki (BMJ 1991; 302:1194). Ethical approval was granted by the Lancaster University Ethics Committee.

### Procedure and measures

The procedure was carried out across two sessions, approximately one week apart from each other (*M* = 6.52 days; *SD* = 3.55 days). This strategy was adopted in order to accommodate infants’ reduced attention span and maximize attention to the stimuli. Importantly, the option for two separate sessions minimized the potential carry over effects from one to the other.

#### EEG recording (Session 1)

Stimuli and procedure: The stimuli consisted of audio-video recordings of a peer infant crying and of a peer infant laughing, adapted from Geangu *et al*.^29^. The infants depicted in the stimuli were 8- to 9-months-old at the time of the recording. Each video recording had an average sound intensity of 70 dB and duration of 90 seconds. Stimuli were presented at a size of 24 × 16 cm on 17-inch CRT computer monitor using MATLAB R2012b (MathWorks, Natick, MA). The order of presentation of the stimuli was counterbalanced across participants. The stimulus presentation began with the display of a dynamic non-social animation for capturing infants’ attention to the screen, which varied in duration from participant to participant depending on how attentive they were. Whenever the experimenter judged that the participants were attentively watching the screen, the first stimulus was presented. Between the first and second stimulus, a non-social animation was always displayed with a duration varying randomly between 30 and 60 secs. If infants became distressed during the stimulus presentation, a maximum of 30-sec was allowed for spontaneous recovery before the procedure was stopped and mothers were invited to comfort their infants. During the entire session, infants sat on their mother’s lap at a distance of approximately 70 cm from the monitor in a dimly lit room. In order to minimize the possibility that mothers could influence infants’ responses to the stimuli, mothers were instructed not to interact with their infant (e.g., talk with, draw attention to the stimuli, display emotional expressions). Cases where these instructions were not followed, were excluded from further analysis. Mothers were told that they can, and should, prevent the infants from grabbing the net/electrodes, and to hold them in a relatively stable position as much as possible throughout the testing session.

EEG recording and analysis: EEG was recorded continuously using a 128-electrode HydroCel Geodesic Sensor Net (Electrical Geodesic Inc., Eugene, OR) and amplified using an EGI NetAmps 300 amplifier. On-line recordings were referenced to the vertex electrode (Cz), and then off-line re-referenced to an average reference. The signal was band-pass filtered at 0.1–100 Hz. EEG data were digitized online at a sampling rate of 500 Hz per channel. Electrode impedances were checked prior to the beginning of the recording and considered acceptable if lower than 50 KΩ, which is a conservative threshold for infants and in accordance with the methodological recommendations for this age group^58,59^. EEG data were further processed offline using NetStation v4.6.4 (Eugene, OR). EEG data were band-pass filtered (0.3–30 Hz), and segmented according to emotional condition (cry stimulus and laughter stimulus), arising 1.5-min epochs for each task for participant. Next, the segments were checked through visual inspection for eye-movements, eye-blinks and other body movement artifacts. Segments with more than 8 bad channels (besides the 11 marked as bad eye-leads) were manually rejected. For the remaining segments, individual bad channels were replaced using spherical spline interpolation. EEG data were then processed in Matlab R2012b (Mathworks Inc., Natick, MA) for artifact rejection and power analysis. EEG segments showing amplitudes greater than +/−175 µV were marked as bad. The remaining artifact-free segments were analyzed with a Fast Fourier transform (FFT) with a 1-sec Hanning window and 50% overlap with a frequency bin of 0.25 Hz. Participants with less than 10 artifact-free epochs were removed from further analysis to ensure a stable estimate of alpha activity (N = 4). Another 4 participants were removed from further analysis because the EEG recording was stopped early due to their distress, failing to provide sufficient data points. The final sample had an average of 67.53 epochs (*min* = 10, *max* = 179) in the laugh and 70.41 epochs (*min* = 11; *max* = 156) in the cry conditions. Absolute power spectral density (psd) values for each segment were computed for the 5–7 Hz frequency band for three reasons: (1) majority of the EEG power was represented within this frequency band^60^; (2) previous studies have associated this frequency with emotion reactivity and emotion regulation during infancy^61–63^; (3) it showed the greatest sensitivity to asymmetry (see Supplementary Information). Alpha power spectral density values were analyzed after being natural log (ln) transformed to normalize the distribution.

Frontal asymmetry scores for each infant in each condition (cry video - ASYM_cry_; laugh video - ASYM_laugh_) were obtained by subtracting the left frontal hemisphere (F3) log-transformed alpha power from the right frontal hemisphere (F4) log-transformed alpha power values (i.e., ln(F4) − ln(F3)). Therefore, positive scores correspond to greater alpha power in the right hemisphere (or increase left activity interpreted as approach-oriented activity) while negative scores correspond to greater alpha power in the left hemisphere (or increased right activity interpreted as withdrawal-oriented activity).

**Figure 1:**
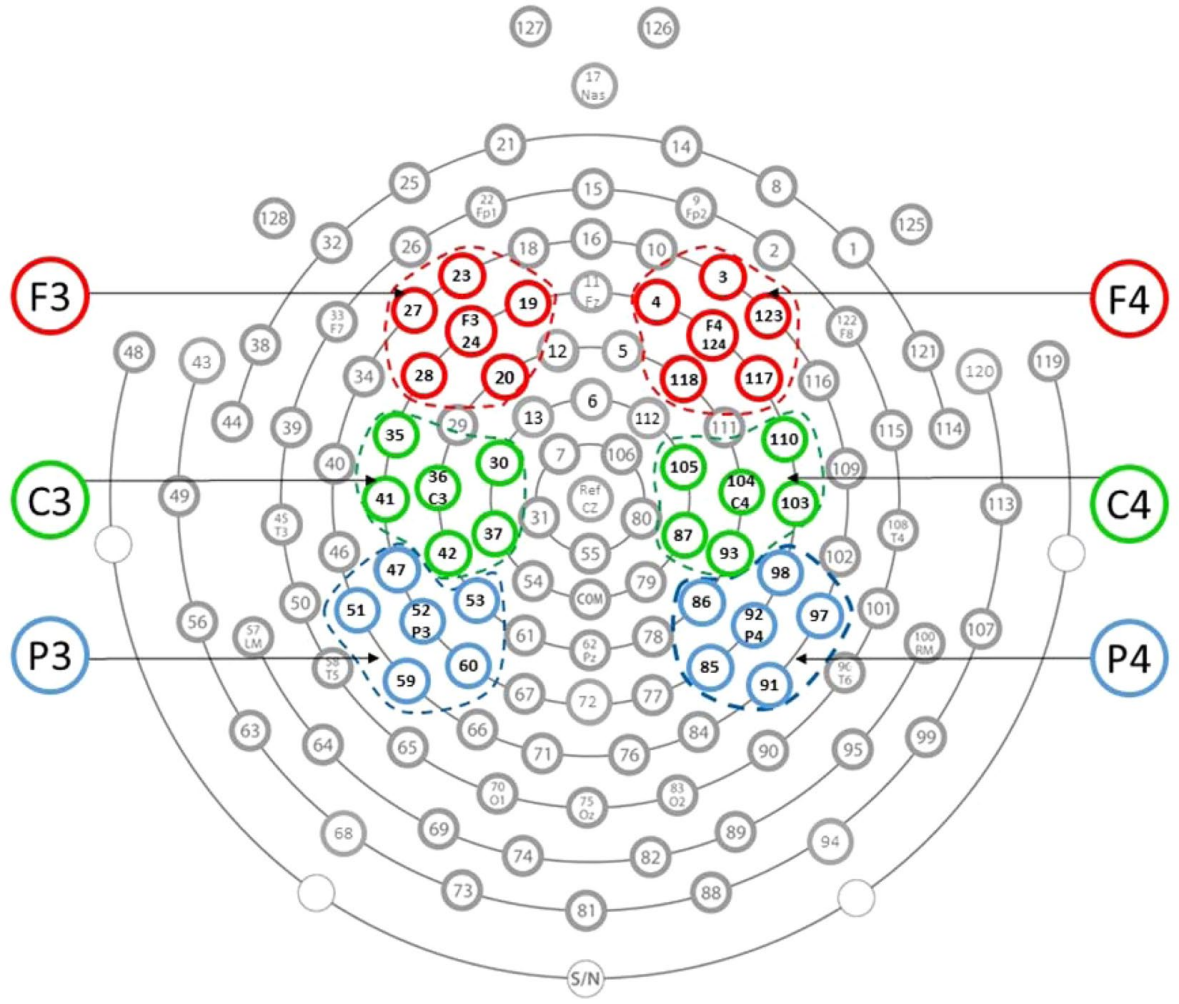
The clusters of electrodes from the 128-electrode HydroCel Geodesic Sensor Net included in the analysis: F3 (19, 20, 23, 24, 27, 28); F4 (3, 4, 117, 118, 123, 124); C3 (30, 35, 36, 37, 41, 42); C4 (87, 93, 103, 104, 105, 110); P3 (47, 51, 52, 53, 59, 60); P4 (85, 86, 91, 92, 97, 98).

In order to establish that the results for the alpha asymmetry were specific to the frontal locations, alpha power spectral density values were also derived from central (C3/C4) and parietal (P3/P4) scalp locations (see Fig. 1 for the clusters of electrodes corresponding to the scalp locations included in the analysis).

#### Behavioral recordings (Session 2)

Stimuli and procedure: The stimuli were similar to those used in Session 1, although they differed with respect to length (120 seconds) and the identity of the infant displaying crying and laughing. Again, the infants depicted in the stimuli were 8- to 9-months-old at the time of the recording. The video recordings were sourced from a professional online database (www.istockphoto.com) and were edited to the required duration and an average sound intensity of 70 dB. Each stimulus was displayed at 43 × 27 cm on a 17″ computer monitor. The procedure began with the presentation of a non-social attention grabber, to ensure that the participants were attending to the screen. During the entire procedure, the infant was seated in an age appropriate chair, at the same height and approximately 70 cm away from the screen. The participants’ behavior was recorded by 4 cameras, three located in corners of the room and one placed above the monitor, allowing a close view of the face. Between the stimuli, a 180 seconds break was introduced, during which the experimenter came back to the room and played with the infant. During the break, an animation film was played on the screen. For the entire duration of the session, the mothers were instructed to sit approximately 2 meters behind the infant, reading a magazine, and without engaging through eye contact or voice with the infant.

Behavior coding criteria: We were interested in overt responses indicative of approach behaviors and emotional reactivity. Based on previous research^27,64–66^ and a preliminary inspection of the recordings, the following responses were coded: (a) negative emotional vocalizations; (b) negative facial expressions; (c) positive emotional vocalizations; (d) positive facial expressions; (e) emotionally neutral vocalizations; (f) approach behaviors; (g) withdraw behaviors; and h) looking time. The coding criteria were derived from the Laboratory Temperament Assessment Battery^67^ (Lab-TAB). In terms of negative facial expressivity, our aim was to capture facial responses which may suggest that infants respond to their peers with emotionally congruent expressions. Thus, we opted for a more generic category, which includes displays of anger, sadness, and fear, as they might be present during infant cry. Table 1 provides a detailed description of the coding criteria for each type of response. A second observer coded 20% of the recordings for reliability (intraclass correlation coefficient for absolute agreement, the reliability coefficients are presented in Table 1).

**Table 1:** Coding criteria for the responses during Session 2.

Response	Coding Criteria
Looking time (0.981)	The duration of visual fixations towards the stimulus. NOTE: Blinks were considered as part of a continuous fixation to the stimulus.
Positive Facial Expression (0.973)	Specific movements in both of the following face regions should be displayed. *Upper Face* (eyes, brows, forehead): eyes are squinted or do not change, furrow below the eyes deepens. *Lower Face*: cheeks are raised, lip corners are raised (either unilaterally or bilaterally). Note: when a brow movement originated in eye/head movement (e.g., infant looking up/down), the action was not coded as brow movement.
Negative Facial Expression (1.000)	Specific movements in both of the following face regions should be displayed. *Upper Face* (eyes, brows, forehead): inner corner of the eyebrows are lowered and drawn together resulting in furrows between the eyes, inner corners of the eyebrows are raised and drawn together resulting in furrows in the middle of forehead, squinted eyes, furrow below the eyes deepens. *Lower Face*: Wide-opened square mouth specific for cry, sad pout, lips pressed together. Note: when a brow movement originated in eye/head movement (e.g., infant looking up/down), the action was not coded as brow movement.
Positive Vocalizations (0.976)	Any vocal production that can be identified as being positively toned, including laughter, babbling with positive prosody, and squealing.
Negative Vocalizations (1.000)	Any vocal production that can be identified as being negatively toned, including whimpering, whining, mild protest, cry/scream.
Neutral Vocalizations (0.982)	Any vocal production that cannot be evaluated as having either positive or negative emotional intonation (e.g., emotionally neutral babbling).
Approach (0.976)	Changes in the upper body position which reduce the distance between the participant and the screen. In order to be coded as approach, these responses need to be associated by visual engagement with the stimulus.
Withdraw (0.978)	Changes in the upper body position which increase the distance between the participant and the screen. Attempts to escape from the chair, including turning away, leaning away, arching back, or twisting in the chair were also coded as withdraw behaviors. Some of these behaviors may be associated with visual disengagement from the stimulus, although this was not mandatory. Head turning in the absence of the upper body turning away was not coded as withdraw behavior.

For coding purposes, all video recordings were divided in 10-sec units. Some of the responses (a–g) were coded as present or absent for each unit. In order to account for variations in stimulus duration length caused by participant’s emotional state, a percentage of units with response present was calculated from the total number of units coded for each participant. For looking time, we coded the duration of visual fixations towards the screen for the entire stimulus presentation. In order to account for variations in stimulus duration, a percentage looking time was calculated out of the entire duration of the stimulus presentation. Datavyu 1.3 free source software (http://datavyu.org) was used for coding. Nine infants were removed from the final dataset for Session 2 due to excessive movement which prevented appropriate face coding (N = 2); technical errors (N = 3); or fussiness at the beginning and throughout the session (N = 4).

Data analysis strategy: The aims of the study were two-fold: (1) to analyze the frontal EEG alpha power (Session 1) and the behavioral responses (Session 2) of 8-month-old infants to their peers’ positive and negative affective states; and (2) to analyze the relation between these responses. In order to ensure that the results for each of these aims rely on the most representative part of our sample, we included in different sections of the analysis all participants who provided analyzable data for that section: Session 1, N = 32; Session 2, N = 22; Session 1 & 2, N = 18. Thus, some of the participants contributing data to either Session 1 or Session 2 analyses, did not contribute data to the analysis of the relation between frontal EEG alpha power and the behavioral responses (Session 1&2).

## Results

### Frontal EEG asymmetry results (Session 1)

**Figure 2:**
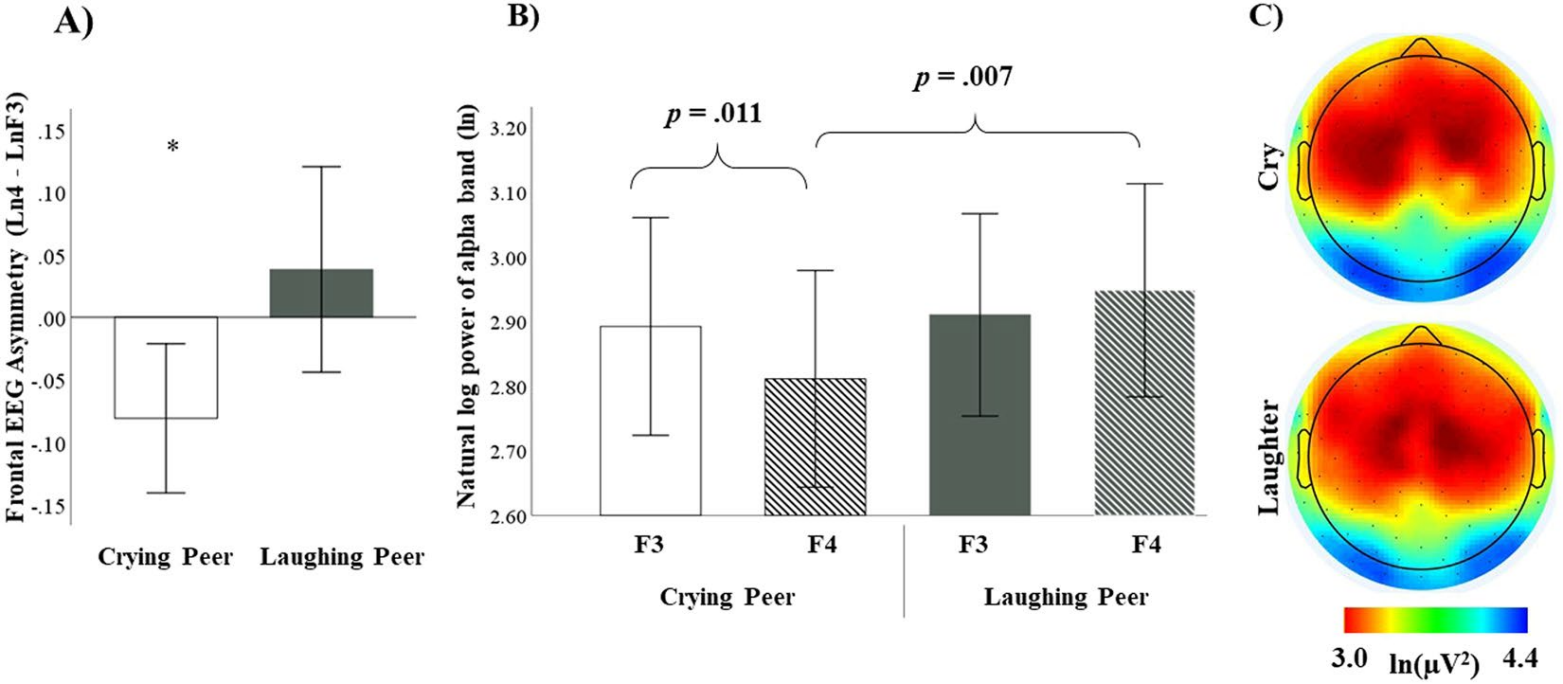
(**A**) Means and standard errors for frontal alpha asymmetry scores collected during the two affective conditions. (**B**) Means and standard errors for the EEG ln alpha power (5–7 Hz) recorded at frontal sensors F3 (left) and F4 (right) during the two affective conditions. (**C**) Scalp wide alpha power for each condition. Note: EEG power is inversely related to cortical activity - high power reflects lower activity. *p < 0.05.

In order to analyze whether peer’s emotions elicited asymmetric frontal EEG activity, separate one-sample t-tests were performed on the frontal EEG asymmetry score obtained during each condition (i.e., laughing and crying). Observing a peer crying elicited an increased left relative to right absolute alpha power (*M* = −0.081, *SD* = 0.168), which was significantly different from zero (*t(31)* = −2.714; *p* = 0.011). Observing a peer laughing elicited some increased right relative to left absolute alpha power (*M* = 0.038, *SD* = 0.232), but the difference from zero did not reach statistical significance (*p* = 0.361; Fig. 2A).

In order to disentangle the separate contributions of the absolute alpha power recorded from the left and right hemisphere to differences in the frontal asymmetry scores recorded for each condition, a 2 (Condition: laughing, crying) x 2 (Hemisphere: right, left) within-subjects ANOVA was performed on the log-transformed alpha power values. A significant Condition × Hemisphere interaction was obtained (*F(1,31)* = 11.166; *p* = 0.002; *η*^2^ = 0.265). Post-hoc pairwise comparisons showed that when infants were exposed to a peer crying, higher absolute alpha power was recorded in the left (*M* = 2.892 μV; *SE* = 0.084 μV) compared to the right hemisphere (*M* = 2.811 μV; *SE* = 0.084 μV), *p* = 0.011). Moreover, exposure to a laughing peer elicited higher absolute alpha power (*M* = 2.948 μV; *SE* = 0.082 μV) in the right hemisphere compared to when participants observed a crying peer (*M* = 2.811 μV; *SE* = 0.084 μV), *p* = 0.007. All other comparisons were not significant (*p* > 0.361; Fig. 2B).

We further compared both central and parietal regions with a 2 (Condition: laughing, crying) × 2 (Hemisphere: right, left) within-subjects ANOVAs on the log-transformed alpha power values to identify whether there were broader effects of emotional condition on alpha asymmetry. Neither central nor parietal regions showed any significant main effects or interactions (*p*s > 0.247) with emotional condition (Fig. 2C).

### Behavioral results (Session 2)

Table 2 provides an overview of infants’ responses to their peer emotions (N = 22). Repeated measures ANOVAs were conducted in order to analyze the differences between the stimuli in terms of facial and vocal expressivity. Greenhouse-Geisser corrections were applied whenever the assumptions of sphericity were violated. For looking time, approach and withdraw behaviors, repeated measures t-tests were performed. All tests were interpreted at a significance threshold of *p* = 0.05.

**Table 2:** Descriptive statistics for infants’ behavioral responses during Session 2 and the results of comparisons between stimuli (N = 22).

	Peer Crying			Peer Laughing			*p*
	*M*	*SE*	%	*M*	*SE*	%	
Looking time^a^	68.88	4.16	NA	59.80	3.73	NA	0.02
Vocalizations^b^							
Negative	24.62	8.12	50.0	6.16	2.74	27.3	0.01
Positive	3.79	2.10	18.2	6.44	2.68	31.8	0.43
Neutral	18.18	5.10	59.1	20.90	5.05	54.5	0.57
Facial expressions^b^							
Negative	21.21	7.61	45.5	6.29	3.28	27.3	0.02
Happy	11.36	3.36	50.0	28.74	6.27	72.7	0.00
Approach^b^	24.62	5.04	72.7	27.55	6.19	68.2	0.61
Withdrawal^b^	51.52	6.41	91.9	47.74	5.33	95.5	0.52

*Note*. % Refers to the percentage of infants displaying the behavioral response; ^a^Percentage of absolute duration from the stimulation duration; ^b^Percentage of 10-seconds units for which the behavior was present from the total number of units.

The 2 (Condition: Cry, Laughter) × 2 (Emotion: Positive, Negative) repeated measures ANOVA for facial expressivity revealed a significant interaction between stimulus and emotion, *F(1*,*21)* = 16.391; *p* = 0.001, *η*^2^ = 0.438. Post-hoc pairwise comparisons indicated that infants responded with more negative facial expressions to the crying peer than to the laughing one, and with more positive facial expressions to the laughing peer than the crying one (Table 2). Also, infants displayed more positive than negative facial expressions while observing the laughing peer (*p* = 0.008). No other significant differences were observed (*p* > 0.304). The 2 (Condition: Cry, Laughter) × 3 (Emotion: Positive, Negative, Neutral) repeated measures ANOVA for vocal expressivity revealed a significant main effect of condition, *F(1*,*21)* = 7.727; *p* = 0.011, *η*^*2*^ = 0.269, which was qualified by a significant interaction with emotion, *F(1.31, 21)* = 4.724; *p* = 0.030, *η*^*2*^ = 0.184. Post-hoc pairwise comparisons showed that infants manifested more emotionally negative vocalizations while observing the crying peer than the laughing one. Observing the crying peer also elicited more negative (*p* = 0.029) and neutral (*p* = 0.015) vocalizations than the positive ones. In response to the laughing peer, infants manifested more emotionally neutral vocalizations than negative (*p* = 0.007) and positive (*p* = 0.021) ones. No other significant differences were observed (*p* > 0.432).

Infants looked longer at the crying than at the laughing peer, *t(21)* = 2.449; *p* = 0.023. No significant differences between the stimuli emerged for the approach and withdraw behaviors.

### Relation between frontal EEG asymmetry and behavioral responses to peers’ emotions (Session 1&2)

In order to test our predictions about the relation between individual variability in the lateralization of the frontal EEG alpha power and the behavioral manifestations of approach/withdrawal and emotion expressivity, Pearson’s correlations between the frontal EEG asymmetry scores (Session 1) and the behavioral responses (Session 2) to peers’ emotions were performed (N = 18). The performed correlations were informed by previous research^51,52,54,57,68^ investigating the link between infant asymmetric frontal alpha power and behavioral responses to emotional events. Thus we did not correct for multiple correlations which might obscure expected results and lead to Type II errors^69,70^.

**Figure 3:**
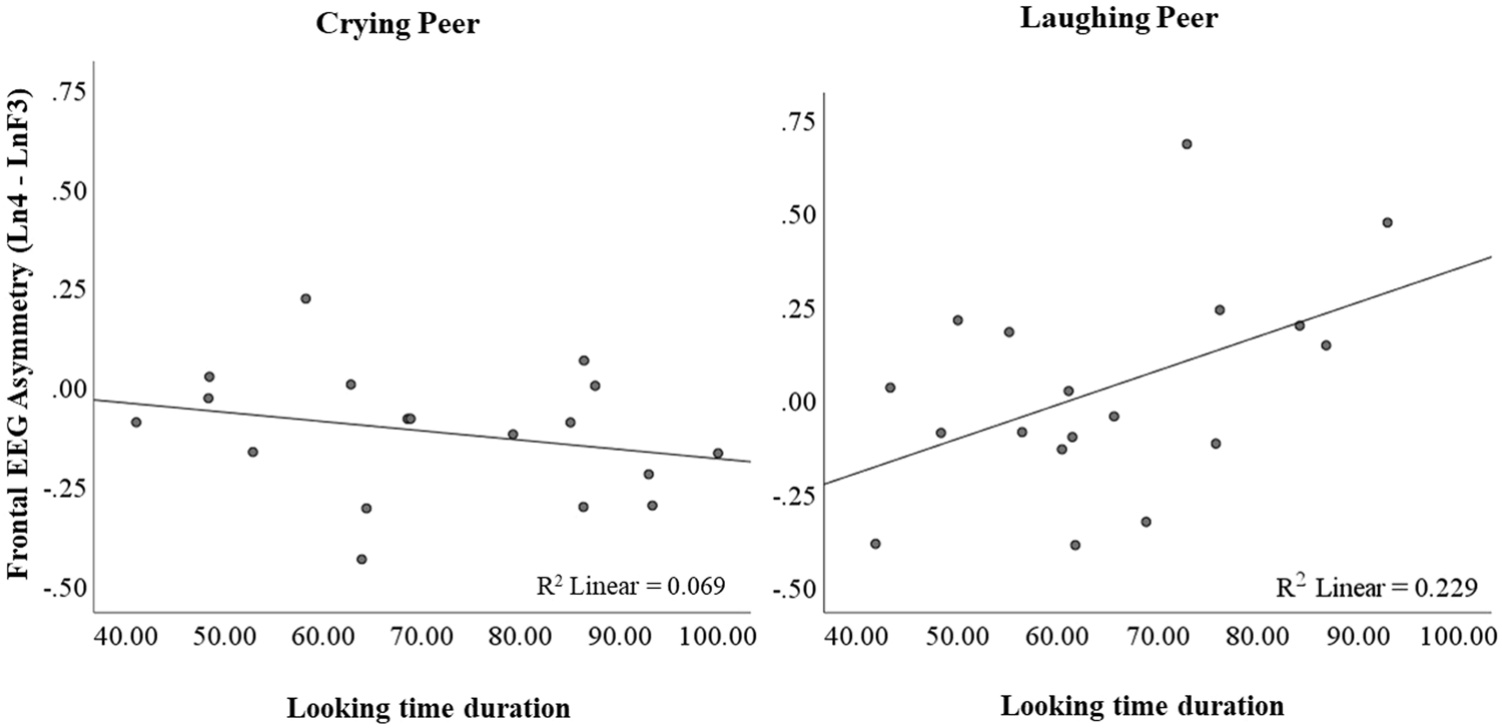
Correlation between frontal EEG asymmetry observed in infants during the presentation of video-films of a peer crying (**A**) and a peer laughing (**B**) with their looking time scores.

Infants’ frontal asymmetry scores recorded in response to the peer crying (i.e., cry lnF4-lnF3 = ASYM_cry_) were positively correlated with the with the approach behaviors (*r* = 0.653; *p* = 0.003) displayed when watching a peer crying in the second session. This indicates that infants who displayed greater degree of left frontal asymmetry when watching a peer crying exhibited more approach behaviors when exposed to the peer crying film in the second session. Infants’ frontal EEG asymmetry scores recorded in response to the laughing peer (i.e., laughter lnF4-lnF3 = ASYM_laugh_) were positively correlated with the amount of time infants looked at a happy peer (*r* = 0.478; *p* = 0.045) in the second session (Fig. 3B). That is, infants who exhibited more left frontal asymmetry during the laughter film spent more time looking at a peer laughing. Additionally, ASYM_laugh_ was significantly correlated with the proportion of positive vocalizations (*r* = 0.519; *p* = 0.027) and marginally correlated with the neutral vocalizations (*r* = 0.463; *p* = 0.053) emitted in response to the sound of a peer laughing. Greater degree of left frontal asymmetry was linked to greater emission of neutral vocalizations during the laughter condition. No other significant correlations between the frontal EEG asymmetry scores (Session 1) and the behavioral responses (Session 2) to peers’ emotions were found (*p* > 0.145).

## Discussion

The current study aimed to investigate the pattern of infant asymmetric frontal EEG alpha power in response to peers’ affective states, and its relation to simple behavioral manifestations of social approach and disengagement. Towards these aims, we presented 8-month-old infants with video recordings of other infants laughing and crying, while we recorded frontal EEG alpha power and examined their behavioral responses during a separate session. The results show that observing peers’ positive and negative affective states elicit distinct patterns of asymmetric EEG alpha power and behavioral manifestations in 8-month-old infants.

At the behavioral level, 8-month-old infants’ emotional responses tended to converge with the affect displayed by their peers. Importantly, this emotional convergence was recorded not only in response to peers’ negative affect as previously shown by several studies^24,27,30,38^, but also for manifestations of happiness. Observing a distressed infant lead to more displays of negative affect in face and voice than when watching a laughing infant. In turn, peer laughter elicited more facial expressions of happiness. We recorded these behaviors in a separate session from the EEG one, where infants attended independently to the stimuli (i.e., not held by their mothers and not in the immediate proximity of an adult) and had more possibilities to move compared to the EEG session. Certainly, these only represent approximations of their real encounters with peers. Nevertheless, similar video recordings of peer affect were shown to elicit sympathetic arousal in infants as reflected by changes in pupil diameter^28,29^, suggesting good ecological validity. Moreover, infants in our study appeared to be interested in the stimuli as they engaged visually with them for more than half of their duration. Although they did so for both emotional expressions, they tended to look more at the crying infant.

In line with current theoretical models and previous investigations of infant neural responses to emotional social and non-social stimuli^45–52,55^, our study shows that 8-month-old infants manifested frontal EEG alpha activity lateralized to the left hemisphere when observing their peers’ negative affect. Since alpha activity has an inhibitory influence on the cortical activity, increased left relative to right frontal alpha power suggests higher activity of the right frontal cortex^45,47^. This means that infants not only show increased right frontal cortex activity when they encounter a distressed adult^52,54,55,71^, or when they perceive cues of social threat (i.e., an adult stranger^51,72^; a social agent blocking the goal achievement of another^39^), but also when they see a crying peer. According to an approach-withdrawal motivational model of frontal asymmetry^45,48,49^, these findings suggest that 8-month-old infants are in general more likely to withdraw in the presence of a peer in distress. Nevertheless, variations in the asymmetric frontal EEG alpha power in response to the crying peer were also observed, and these tended to relate to infants’ behavioral responses.

Infants who were more likely to respond with reduced right and greater left frontal asymmetry to crying were also more likely to physically approach their distressed peer. Within the theoretical framework proposing the involvement of the left frontal cortex in an approach system responsible for goal-directed behaviors^46,48,49^, these results suggest that 8-month-old infants who are more motivated to act are also more likely to approach a peer in distress. Interestingly, the asymmetric frontal activity was not related to the expressions of negative affect when watching a peer crying. These results could partially indicate a dissociation between the motivational and affective dimensions of 8-month-old infants’ responses to their distressed peers, similar to what has been previously reported in adults^16,49,73^. For example, the activation of the brain regions functionally linked to the motivation to act and not those typically associated with experiencing emotions predicted how much adults behave prosocially during everyday life^16^. The interactions between infants become increasingly reciprocated from 6- to 9-months, relying on simple motor acts, such as moving towards and touching their peer^30,35^. Importantly, these simple motor acts continue to be present during toddlerhood and preschool years, when they are related to complex prosocial behaviors, such as helping and comforting, as well as measures of emotional and cognitive dimensions of empathy^17,18^. Taken together, these findings argue in favour of the need to study infants’ approach behaviors towards others in distress as potential origins of the mature forms of prosocial behaviors. Further longitudinal studies are needed to test this proposal. Concurrent measures of frontal asymmetry, emotional expressivity, and social approach behaviors at multiple time points beginning with infancy could be particularly useful in this respect.

Contrary to some of the previous findings that infants respond with increased right lateralized frontal EEG alpha power to adult facial and vocal expressions of happiness^54,55^, but in line with others^74^, at the group level, 8-month-old infants in the current study did not show frontal EEG alpha power asymmetry in response to peer laughter. One possible explanation for these results is that the infants had a generally high degree of positive affect or approach orientation and that the laughter stimuli did not generate any greater left frontal asymmetry from that baseline state. Alternatively, it could be that infants’ ability to process and respond to the communicative value of their peers’ laughter may be insufficiently developed before the age of 12-months^11,28,29^. Although from an early age infants are able to laugh^75^, this emotional expression appears to be more frequently associated with the interactions with adults^76^. As a result, infants may encounter less frequently these specific facial expressions and non-verbal vocalizations when interacting with peers^77,78^, with consequences for the development of their abilities to extract the corresponding social message. Interestingly, those 8-month-old infants who did respond with a more left lateralized frontal activity to the laughing peer tended to be those who looked more at this stimulus during the behavioral session. As looking behavior can reflect perceptual and cognitive processing of the stimulus^79,80^, it could be that the left frontal lateralization is more likely to appear in those infants who attend more, and thus are more likely to extract the relevant emotional information from facial and vocal expressions of laughter^54^. Visual engagement with others could also be regarded as an index of social approach^54^, and from this perspective our findings would indicate a motivational link between 8-month-olds’ tendency towards left frontal asymmetry and approaching behaviors towards a happy peer. Due to the correlational nature of the analysis, it is, however, difficult to draw conclusions in this respect. The inclusion of a non-emotionally valenced baseline, larger sample size and a wider age range, could allow in the future a more comprehensive analysis of the relation between the cognitive processing of emotional information and frontal asymmetry in response to peers, as well as the meaning of looking behavior during infant interactions.

Another possible explanation for the pattern of frontal asymmetry in response to peer laughter could be that, although observing peers’ happiness leads to some positive affect in 8-month-old infants as shown by their facial expressivity during the behavioral session, this may only reflect limited sympathetic arousal. Indeed, before the age of 12 months the pupillary dilation response to peer laughter is brief and reduced compared to that for crying^28,29^. In a similar vein, previous studies have shown greater left lateralized frontal activity when 2-month-old infants show intense facial expression of happiness, but not when these were less intense^52^, while in adults, pleasant pictures tended to elicit increased left frontal activity only if a propensity to experience positive affect was already present^81,82^. The inclusion of concurrent measures of frontal asymmetry, arousal, and emotional expressivity would be useful for testing this proposal in future research.

Important to note are some limitations to the current study. Although we included a larger sample of participants to begin with, only a subset completed both testing sessions. We are confident, however, that our results are not due to the sample size. First, our findings converge in several ways with those previously reported in studies using similar paradigms^24,26,54,55,72^ and show medium-large to large effects. Second, the correlations fall within the 95% CI (*r*s = 0.30 to 0.52) of the correlation coefficients reported in previous studies that investigated the relation between infant frontal asymmetry and behavioral responses similar to those investigated here^54,57,68,83^. This suggests that our study is sufficiently powered to detect correlations between brain and behavior^84,85^. The recording of the behavioral and frontal EEG during separate testing sessions may also limit the interpretation of our findings. The greater freedom of movement allowed during the behavioral session could have caused too much noise in the EEG data and was the main reason behind our procedural decision. Although recent studies show that the patterns of frontal alpha power asymmetry in response to emotional events are stable across measurements at different time points in infancy and toddlerhood^56^, it would be important in the future to test the possibility of using concurrent behavioral and brain activity measures, in order to assess the generalizability of our results. Furthermore, the delineation between the frontal alpha and the theta band for the lower frequencies in infants younger than 10-months appears to be still open for discussion and further validation. For example, while some previous studies include some of the lower frequencies (e.g., 4 Hz) in the frontal alpha band^61–63^, others do not^86^. Based on preliminary analysis of our results (see Supplementary Information), as well as based on previous studies using similar age groups and experimental paradigms^61–63^, we considered the 5 to 7 Hz band is most representative of the frontal alpha. Nevertheless, further validation using emotional stimuli is needed^60^.

In summary, the results show that observing other infants crying or laughing elicits in 8-month-old infants distinct patterns of asymmetric frontal activity, as well as overt responses suggesting the presence of convergent emotional responses and social approach behaviors. These findings add valuable information to a limited body of knowledge about the potential early origins of empathy and pro-social behaviors, and their underlying neurocognitive mechanisms. The specific link between approach behaviors and variations in left frontal activity indicates the presence of a motivational dimension to infants’ responses to distressed peers and emphasizes the importance of investigating the role of these simple behaviors in the ontogeny of prosocial abilities.

## Electronic supplementary material

Supplementary Information

## Data Availability

The datasets generated during and/or analyzed during the current study are available from the corresponding author on reasonable request.
