# Supplementary material for: Eight-month-old infants’ behavioral responses to peers’ emotions as related to the asymmetric frontal cortex activity
Source: Sci Rep. 2018 Nov 21;8:17152. doi: 10.1038/s41598-018-35219-4 (PMC6249297; doi:10.1038/s41598-018-35219-4)
Supplement: Supplementary file 1 — Supplementary Information [file 41598_2018_35219_MOESM1_ESM.docx]

Eight-month-old infants’ behavioral responses to peers’ emotions as related to the asymmetric frontal cortex activity.

Maria M. Crespo-Llado, Ross Vanderwert, Elisa Roberti, and Elena Geangu

**Supplementary Information**

In order to select the frequency range for the alpha band, we followed the recommendations suggested in the literature (Marshall et al., 2002). The distribution of the relative power spectra for each infant between 2Hz and 12Hz at occipital electrodes (Supplementary Figure 1) initially suggested that the peak alpha frequency ranges from 4 to 7Hz.


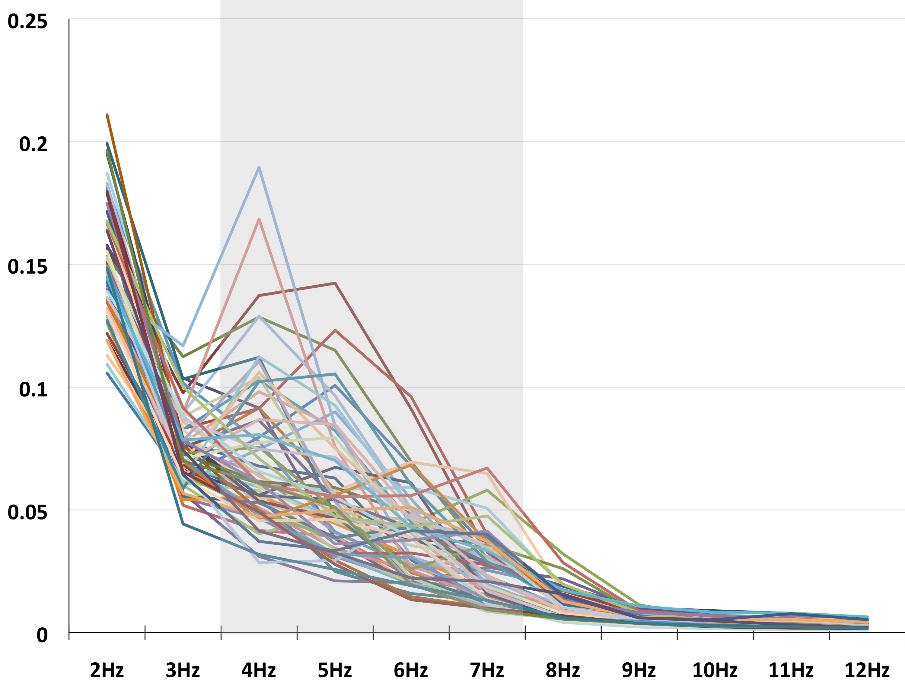


Supplementary Figure 1: Relative power spectra for each infant between 2Hz and 12Hz at occipital electrodes. Each line represents a separate infant.

A 2 (Condition: laughing, crying) x 2 (Hemisphere: right, left) within-subjects ANOVA was performed on the log-transformed 4-7Hz power values. A significant Condition x Hemisphere interaction was obtained (*F(1,31)* = 5.787; *p* = .022; *η^2^* = .157). Post-hoc pairwise comparisons showed that when infants were exposed to a peer crying, higher absolute 4-7Hz power was recorded in the left (*M* = 3.513 μV; *SE* = .086 μV) compared to the right hemisphere (*M* = 3.436 μV; *SE* = .086 μV), *p* = .020. Moreover, exposure to a laughing peer elicited higher absolute 4-7Hz power (*M* = 3.518 μV; *SE* = .083 μV) in the right hemisphere compared to when participants observed a crying peer (*M* = 3.436 μV; *SE* = .086 μV), *p* = .047. All other comparisons were not significant (*p* > .391; Supplementary Figure 2A).


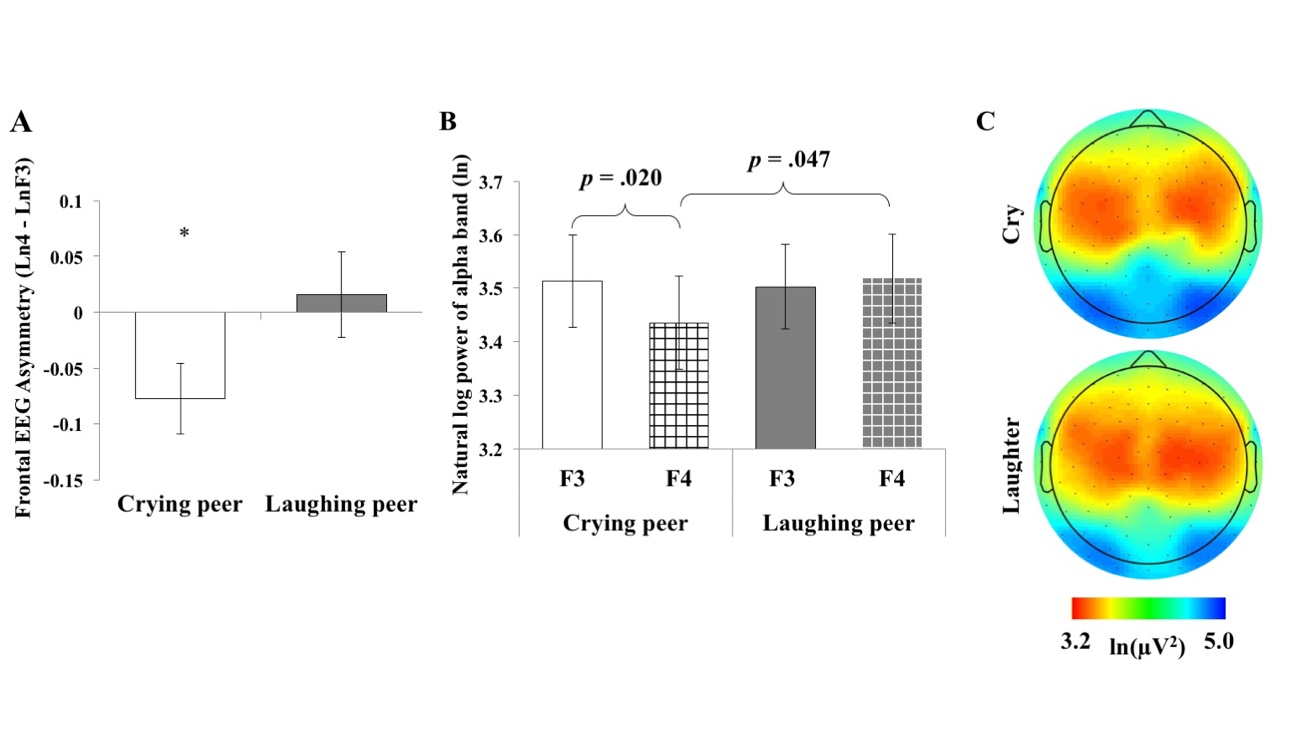


Supplementary Figure 2. (A) Means and standard errors for the EEG ln (4-7Hz) power recorded at frontal sensors F3 (left) and F4 (right) during the two affective conditions. (B) Scalp wide (4-7Hz) power for each condition. Note: EEG power is inversely related to cortical activity - high power reflects lower activity. * p < 0.05


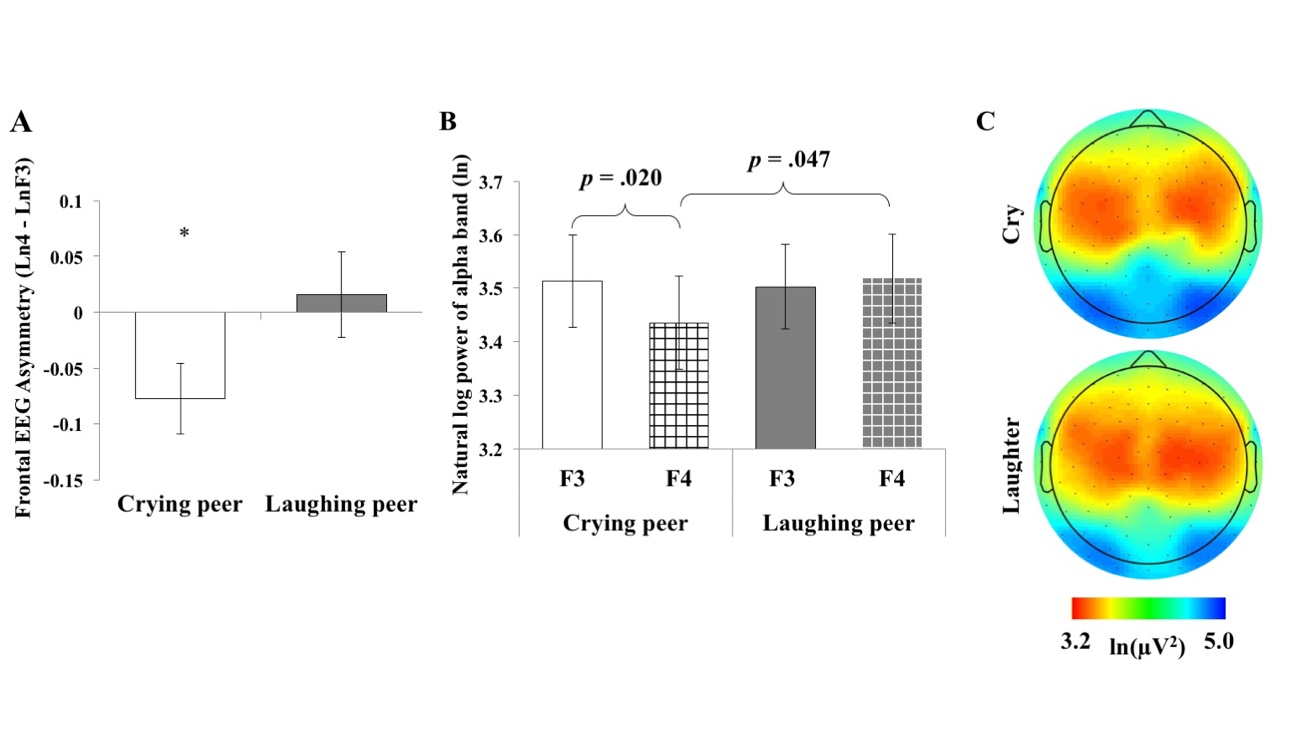


**(A)**

**(B)**

Nevertheless, disagreement exists in the literature with respect to the inclusion in the alpha band of the lower frequencies, particularly the 4Hz, which can be regarded to reflect theta activity (e.g., Marshall et al., 2002; Bazhenova et al., 2007; Calkins, Fox, & Marshall, 1996; Santesso, Schmidt, Trainor, 2007). As a consequence, we analysed the asymmetry effects for each single-hertz bin between 4Hz and 8Hz using a 2 (Condition: laughing, crying) x 2 (Hemisphere: right, left) within-subjects ANOVA (Supplementary Table1). The results showed that the frequency bins between 5 and 7Hz demonstrate the greatest asymmetry, which motivated us to focus on this band for the analysis included in the main manuscript.

Supplementary Table 1. The results of the 2 (Condition: laughing, crying) x 2 (Hemisphere: right, left) within-subjects ANOVA for each single-hertz bin between 4 and 8 Hz.

| **Frequency bin** | **Condition** | | | **Hemisphere** | | | **Condition x Hemisphere** | | |
| --- | --- | --- | --- | --- | --- | --- | --- | --- | --- |
|  | *F_(1,31)_* | *p* | *η^2^* | *F_(1,31)_* | *p* | *η^2^* | *F_(1,31)_* | *p* | *η^2^* |
| 4Hz | .219 | .643 | .007 | 1.157 | .290 | .036 | .616 | .438 | .019 |
| 5hz | 1.304 | .262 | .040 | 1.130 | .296 | .035 | 3.653 | .065 | .105 |
| 6Hz | 6.245 | .018 | .168 | .046 | .832 | .001 | 11.125 | .002 | .264 |
| 7Hz | .087 | .770 | .003 | .024 | .879 | .001 | 9.679 | .004 | .238 |
| 8Hz | .505 | .483 | .016 | .169 | .684 | .005 | .683 | .415 | .022 |
